# Impact of Bisphosphonate Therapy on Oral Health in Patients with Breast and Prostate Cancer and Bone Metastases: A Comprehensive Study

**DOI:** 10.3390/cancers16061124

**Published:** 2024-03-11

**Authors:** Jacek Calik, Katarzyna Calik, Natalia Sauer, Bogucki Zdzisław, Piotr Giedziun, Jacek Mackiewicz, Marek Murawski, Piotr Dzięgiel

**Affiliations:** 1 Department of Clinical Oncology, Wroclaw Medical University, 50-556 Wrocław, Poland; 2 Old Town Clinic, 50-136 Wrocław, Poland; katarzyna.calik@oldtownclinic.pl (K.C.); natalia.sauer@student.umw.edu.pl (N.S.); 3 Department of Clinical Pharmacology, Faculty of Pharmacy, Wroclaw Medical University, 50-556 Wrocław, Poland; 4 Department of Prosthetic Dentistry, Wroclaw Medical University, 50-425 Wrocław, Poland; zdzislaw.bogucki@umw.edu.pl; 5 Faculty of Information and Communication Technology, Wrocław University of Science and Technology Centre for Scientific and Technical Knowledge and Information, 50-370 Wrocław, Poland; piotr.giedziun@pwr.edu.pl; 6 Department of Medical and Experimental Oncology, Institute of Oncology, Poznan University of Medical Sciences, 60-512 Poznan, Poland; jmackiewicz@ump.edu.pl; 7 1st Department of Gynecology and Obstetrics, Wroclaw Medical University, 50-599 Wrocław, Poland; marek.murawski@umw.edu.pl; 8 Division of Histology and Embryology, Department of Human Morphology and Embryology, Wroclaw Medical University, 50-368 Wrocław, Poland; 9 Department of Physiotherapy, University School of Physical Education, 51-612 Wrocław, Poland

**Keywords:** bisphosphonates, stomatognathic system, bone metastases, BRONJ (bisphosphonate-related osteonecrosis of the jaw), oral hygiene

## Abstract

**Simple Summary:**

This study delves into bisphosphonate therapy’s effects on oral health in 80 breast and prostate cancer patients with bone metastases. Bisphosphonates, crucial for managing skeletal complications, are linked to bisphosphonate-related osteonecrosis of the jaw (BRONJ), presenting in 0.8–18.5% of patients with symptoms like pain, tissue swelling, and tooth mobility. Through dental and radiological assessments—including DMFT and OHI-S indices, periodontal measurements, and panoramic X-rays—the research uncovers a notable decline in oral hygiene post-therapy, evidenced by worsened DMFT, OHI-S, PD, and CAL scores. Additionally, radiological changes hint at bisphosphonates’ impact on bone density and structure. Despite no significant changes in TMJ function, the findings underscore the need for ongoing dental care and risk mitigation strategies for BRONJ, emphasizing the vital role of regular dental monitoring and proactive oral hygiene practices in the comprehensive care of cancer patients undergoing bisphosphonate therapy.

**Abstract:**

This study investigates the impact of bisphosphonate therapy on the stomatognathic system in 80 patients with cancer of the breast and prostate with bone metastases. Bisphosphonates are integral for managing skeletal complications in these malignancies but are associated with bisphosphonate-related osteonecrosis of the jaw (BRONJ), affecting 0.8–18.5% of patients. BRONJ manifests with pain, neuropathy, tissue swelling, mucosal ulceration, tooth mobility, and abscesses, yet its pathogenesis remains elusive, complicating risk prediction. The research employed comprehensive dental and radiological evaluations. Dental status was assessed using DMFT and OHI-S indices, Eichner’s classification, and clinical periodontal measurements like the pocket depth (PD), clinical attachment loss (CAL), and modified Sulcus Bleeding Index (mSBI). A radiological analysis included panoramic X-rays for radiomorphometric measurements and TMJ lateral radiographs. Results indicated a significant decline in oral hygiene in patients with cancer after bisphosphonate therapy, marked by increased DMFT and OHI-S scores. Periodontal health also showed deterioration, with increased PD and CAL readings. The incidence of BRONJ symptoms was noted, although exact figures are not quantified in this abstract. The study also revealed changes in radiomorphometric parameters, suggesting bisphosphonates’ impact on bone density and structure. No substantial alterations were observed in TMJ function, indicating a need for extended observation to understand bisphosphonates’ long-term effects on the stomatognathic system. These findings highlight the importance of continuous dental monitoring and prophylaxis in patients undergoing bisphosphonate therapy. Implementing meticulous oral care protocols is essential for mitigating BRONJ risk and managing the complex oral health challenges in patients with cancer.

## 1. Introduction

The development of clinical practice of anticancer medications has revolutionized cancer care over the past thirty years [1]. However, the pursuit of enhanced rates of remission and disease control is accompanied by a concomitant rise in the risk of adverse effects, predominantly affecting the oral cavity and its related anatomical components, including the oral mucosa, salivary glands, jawbones, and cranial nerves [2]. Frequently encountered complications include xerostomia, alterations in taste perception, gingival hypertrophy, and mucosal inflammation stemming from cancer therapy. Additionally, clinical observations reveal the manifestation of supplementary symptoms, such as salivary gland dysfunction, transformations in the oral mucosa, pigmentation anomalies, halitosis, osseous necrosis, opportunistic infections, and hemorrhagic diathesis [3].

Oncological treatment is an expansive concept encompassing not only the eradication of the primary tumor but also a supportive therapeutic approach aimed at mitigating or alleviating the distressing sequelae for the patient. A plethora of these undesirable effects can be either preemptively averted or effectively managed [4]. An illustrative instance of supportive intervention during the course of antineoplastic therapy is the administration of antiemetic agents or agents that bolster marrow function [5,6]. Of notable significance in this regard is the administration of bisphosphonates, which function by impeding osteoclast-dependent bone tissue degradation [7,8]. Bisphosphonates are administered to patients with bone metastases associated with neoplastic diseases [9]. Predominant among these malignancies are prostate and breast cancer [10,11]. While bisphosphonates have been employed in clinical practice for over thirty years, it was only recently that any extended adverse effects on the jawbone were officially documented [12,13,14]. Presently, there is increasing evidence pointing to the impact of bisphosphonates on the development of osteonecrosis in the jaw region (ONJ) [14]. ONJ presents a formidable therapeutic challenge and causes substantial daily discomfort to the afflicted patient. Bisphosphonate-induced osteonecrosis of the jaws (BIONJ) is a condition where bone in the mandible or maxilla remains exposed for an extended period exceeding eight weeks [15]. This occurs in patients who have used or are presently using bisphosphonates and have not undergone jaw radiation therapy.

Regrettably, despite extensive investigation, a definitive patient profile predisposed to this complication remains elusive [16]. The prevailing body of research has yet to provide unequivocal elucidation regarding the etiopathogenesis of aseptic jawbone necrosis linked to intravenous bisphosphonate therapy in the context of breast and prostate cancer and skeletal metastases [17]. The purpose of the research endeavors described herein is to chronicle and assess changes occurring within the distinct components of the stomatognathic system following the initiation of bisphosphonate therapy. This endeavor seeks to identify these changes as potential predictive markers for the development of aseptic osseous necrosis.

The primary objective of this study is to conduct a comparative evaluation of specific components within the stomatognathic system in patients suffering from breast and prostate cancer with bone metastases. We aim to compare these patients before initiating bisphosphonate treatment and six months after its commencement, relative to a control group. Our study’s specific goals encompass a multifaceted assessment. Firstly, we intend to evaluate the dental status and oral hygiene of patients with cancer and skeletal metastases. Additionally, we will examine the condition of the oral mucosa and assess periodontal inflammation in these patients both before and during bisphosphonate therapy. The research will also investigate the occurrence and severity of functional impairments in the temporomandibular joint among patients undergoing bisphosphonate therapy in the context of neoplastic diseases with bone metastases. Furthermore, we will ascertain the influence of tooth loss and the utilization of dental prostheses on manifestations within the stomatognathic system in patients receiving intravenous bisphosphonate therapy for bone metastases. Lastly, the study aims to evaluate jawbone alterations resulting from intravenous bisphosphonate administration in patients with breast and prostate cancer and associated skeletal metastases through radiological assessments. This comprehensive exploration aims to illuminate the intricate implications and clinical considerations associated with bisphosphonate therapy in the realm of cancer metastasis, with a specific focus on its effects on the stomatognathic system.

## 2. Materials and Methods

In pursuit of the predetermined objectives, clinical and radiological investigations were employed. Patients were apprised of the aims and principles of the conducted research and provided voluntary written consent for their participation. Before commencing the study, ethical approval was obtained from the Bioethics Committee in Wrocław under reference number KB-114/2017. Subsequently, consent was secured from the same committee under reference number KB-19/2019.

### 2.1. Characteristics of the Studied Cohorts

The study aimed to evaluate the impact of intravenous bisphosphonate therapy on patients with metastatic breast or prostate cancer affecting the bone. The inclusion criteria for participants were as follows: age between 55 and 85 years, a current diagnosis of breast or prostate cancer with imaging-confirmed bone metastases, eligibility (based on oncological indications and absence of dental contraindications) for intravenous bisphosphonate therapy, no current or past use of bisphosphonate drugs, no history of radiation therapy to the craniofacial region, and written consent to participate in the study.

A total of 234 patients expressed interest in participating. However, 124 patients were excluded from the study due to not meeting the inclusion criteria. Among these, 54 patients (38 men and 16 women) were excluded due to dental contraindications to bisphosphonate therapy initiation, such as the need for tooth extractions, removal of extensive dental calculus, treatment of large periapical lesions or cysts. Thirty-one patients were excluded due to having a cancer type other than breast or prostate cancer. Thirteen patients did not meet the age criterion, and twenty-six were excluded for not meeting other inclusion criteria.

Exclusion criteria during the study were the discontinuation of intravenous bisphosphonate therapy and deterioration of the patient’s general condition, preventing the completion of the study. Based on these criteria, 21 patients were excluded during the study. Additionally, 9 patients did not complete the study due to death.

The comprehensive study encompassed a total of 80 patients, with a mean age of 68.4 ± 8.2, divided into the experimental and control groups. The study population consisted of 60 patients aged 55 to 85 years, undergoing treatment at the Lower Silesian Center of Oncology in Wrocław for breast or prostate cancer. The mean age within this group was 69.6 ± 8.3 (Table 1). Among these, there were 22 female and 38 male participants (Table 2). The control group comprised 20 patients from the Old Town Clinic in Wrocław, aged 55 to 85 years, without neoplastic diseases. This group consisted of 13 females and 7 males (Table 2), with a mean age of 64.9 years ± 7.2 (Table 1).

**Figure 1:**
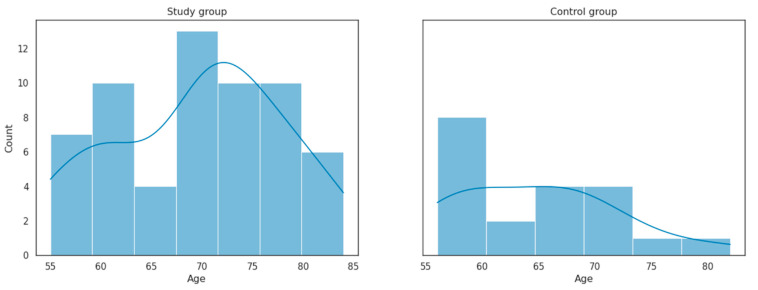
Age distribution in the study group and control group.

The age distribution in the study group and control group is presented in Figure 1.

Each participant underwent dual evaluations comprising both dental and radiological examinations on two visits. Within the study cohort, the initial assessment transpired prior to the onset of intravenous bisphosphonate intervention, followed by a subsequent evaluation six months post-treatment initiation. Conversely, the control group underwent assessments in six-month intervals. The enrollees provided explicit informed consent to partake in the research study and were furnished with an informative pamphlet delineating the study particulars.

### 2.2. A Comprehensive Oral Examination

#### 2.2.1. Subjective Examination

The medical interview encompassed the completion of a health questionnaire, containing not only general information but also inquiries about health-promoting behaviors, hygiene habits, and risk factors associated with avascular necrosis of bone.

#### 2.2.2. Objective Examination

##### Oral Cavity Assessment Using Epidemiological Indices

The evaluation of dental status was carried out under artificial lighting conditions utilizing a dental mirror and a periodontal probe. Results were recorded on a dental diagram, and index values were computed and documented on the patient’s record card. The oral cavity’s condition was evaluated based on WHO criteria, employing the DMFT and OHI-S indices.

The assessment encompassed counting teeth exhibiting decay, extracted teeth, and restored teeth, contributing to the DMFT value.

The presence of plaque and calculus on four buccal surfaces of teeth 16, 11, 26, and 31, and on two lingual surfaces of teeth 36 and 46, was examined. Summing the scores yielded plaque and calculus values (ranging from 0 to 6 points). Subsequently, the obtained score was divided by the number of examined teeth, resulting in the OHI-S value, documented on the individual patient examination card.

The interpretation of OHI-S results relied on ranges established by Greene and Vermilion and is as follows:

0—excellent hygiene.
0.1–1.2—good hygiene.
1.3–3.0—fair hygiene.
3.1–6.0—poor hygiene.

##### Assessment of Dental Gaps

The presence of dental gaps was assessed in all patients according to Eichner’s classification of dental gaps. Dental gaps influence the nature of occlusal contacts and the transmission of occlusal forces between dental arches during mastication. This is intricately related to the function of individual muscles and the stomatognathic system. Scrutiny of mutual occlusal contact arrangement in the dental arches of the examined patients was conducted.

Eichner’s classification, also known as the intermaxillary classification, consists of 3 groups (A, B, C) with numerical subgroups accounting for occlusal conditions.

Furthermore, an assessment of prosthetic reconstructions’ presence, extent, and type, including fixed prostheses, removable mucosal-borne dentures, and mucosal-borne prostheses, was carried out for all patients.

##### Periodontal Status Assessment

For clinical periodontal evaluation, parameters PD, CAL, and mSBI were employed.

The extent of periodontal structure destruction was gauged by measuring the periodontal pocket depth (PD) (in millimeters) and clinical attachment level (CAL) (in millimeters), denoting the distance from the bottom of the periodontal pocket to the cementoenamel junction [18].

CAL was found to be more sensitive to the number of medications taken compared to PD. However, precise differentiation concerning the exact number of medication combinations leading to CAL degradation remains elusive [19].

A scaled periodontal probe (scaled to 11.5 mm), WHO-621, was used to gauge the depth of gingival and periodontal pockets. Each assessed tooth was evaluated at 6 points: mesio-buccal, buccal, distal-buccal, distal-lingual, lingual, and mesio-lingual.

The probe, comprising a handle and a measuring portion, was introduced parallel to the long axis of the tooth during measurement, ensuring constant contact with the root surface down to the pocket’s base. In addition to pocket depth measurements, clinical attachment loss (CAL) was evaluated at the specified points. CAL was calculated by adding the distance between the gingival margin and the cementoenamel junction (CEJ) to the pocket depth.

The summation of all measurements and subsequent division by 6 and the number of teeth yielded average PD and CAL values for each subject.

For inflammation assessment, the modified Sulcus Bleeding Index (mSBI) developed by Mühlemann and Sona [20] was utilized. This index indicates the percentage of bleeding gingival units following gentle probing, according to the formula

mSBI = (sum of bleeding interdental gingival units × 100%)/sum of all examined interdental gingival units(1)

In which interpretation indicates the following:

mSBI > 50%—severe, generalized gingival inflammation.
mSBI 50–20%—moderate gingival inflammation.
mSBI 19–10%—mild gingival inflammation.
mSBI < 10%—absence of gingival inflammation.

Clinical indicators of gingival inflammation include bleeding upon probing of gingival or periodontal pockets [18]. Results of periodontal assessment were documented in the periodontal record form.

##### Mucosal State Assessment

A meticulous evaluation of patients’ mucosal state was conducted, with special attention directed toward prosthetic bases in patients utilizing removable dentures, particularly mucosal-borne prostheses.

##### Temporomandibular Joint Examination

A functional analysis of the stomatognathic system was executed among the examined patients, enabling the assessment of temporomandibular joint dysfunction levels using the Helkimo index. The Helkimo index comprises two components:

Subjective Examination—Based on interviews, the anamnestic index of temporomandibular joint dysfunction (anamnestic index—Ai) was incorporated. The Helkimo anamnestic index (Ai) has three levels:

Ai-0—absence of subjective temporomandibular joint dysfunction symptoms.
Ai-I—slight subjective symptoms, such as noises, clicks, stiffness, or muscular fatigue during mastication.
Ai-II—significant subjective symptoms, including difficulty in wide mouth opening, jaw clenching, pain during movements, facial and jaw area pain, joint dislocation [21].

Clinical Examination—Utilizing the developed examination form and clinical dysfunction index (Di) according to Helkimo, the severity of masticatory system disorders was assessed, encompassing 5 symptoms. The clinical Helkimo protocol involved evaluating jaw movement amplitude during lateral excursions and protrusion, the smoothness and straightness of stomatognathic system movements, the presence of pain during the palpation of masticatory muscles and the jaw area, and during free jaw movements. The sum of points acquired using the clinical dysfunction index (Di) by Helkimo determined the severity of temporomandibular joint dysfunction according to a four-level scale:

Di-0—absence of clinical symptoms: 0 points.
Di-I—minor dysfunction: 1–4 points.
Di-II—moderate dysfunction: 5–9 points.
Di-III—severe dysfunction: 10–25 points [21,22,23,24].

### 2.3. Jawbone Radiological Assessment

Each participating patient underwent a panoramic radiographic examination. Digital panoramic radiographs were taken using the GXDP-300 digital X-ray unit (Gendex Dental Systems, Hatfield, PA 19440, USA) and patient measurements utilized VinWix Platinum v3.3 software (DEXIS, Quakertown, PA 18951, USA) calibrated with 3DSlicer measurements. Patients received panoramic radiographs adhering to the manufacturer’s protocol.

Radiographic images underwent an analysis, involving radiomorphometric measurements (h, MCW), followed by calculating the panoramic mandibular index (PMI). The objective of this analysis was to determine whether dimensional changes occurred in the cortical bone of the mandible due to bisphosphonate use, by comparing mandibular cortical width (MCW) measurements in patients using these medications with measurements on control panoramic radiographs. Devlin and Horner established a correlation between MCW measurement outcomes and bone mineral density in patients [25].

**Figure 2:**
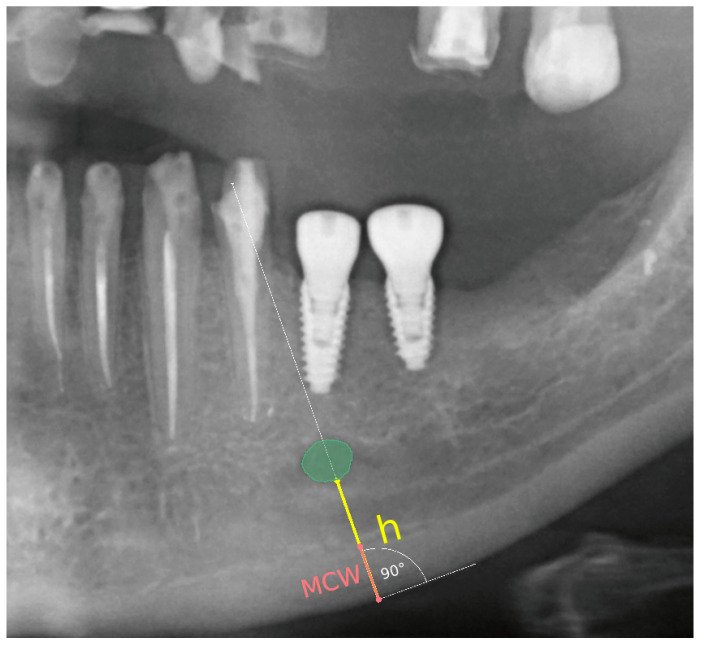
Demonstration of the technique for measuring the compact bone thickness of the mandible and the distance between the mental foramen and the lower border of the mandibular compact bone on pantomographic images using the 3D Slicer software version 4.8.1.

The following radiomorphometric measurements were taken on each radiograph (Figure 2):

A line was established along the lower border of the mandible, and a perpendicular line through the center of the mental foramen was drawn.

Distance between the mental foramen and the lower border of the mandibular cortical bone (h) was measured.

Mandibular cortical width in the chin region (MCW): Thickness of the mandibular cortical bone, measured along a line perpendicular to the lower portion of the mandible through the center of the mental foramen, was determined. A value greater than 3.1 mm was considered normal [26].

Based on these measurements, the panoramic mandibular index (PMI) was calculated. The panoramic mandibular index (PMI) was computed as the ratio of MCW/h [27]. PMI was calculated as the ratio of the thickness of the mandibular corpus compact bone (MCW) to the height measured from the lower border of the mandible to the lower edge of the mental foramen (h). A value greater than 0.3 is considered the norm [28,29].

(2) $$PMI=\frac{MCW}{h}$$

The MCW measurement was obtained by drawing a line tangential to the lower border of the mandible and a second line perpendicular to this tangent intersecting the lower border of the mandible and the mental foramen, along which the thickness of the mandibular cortex was measured.

All measurements were performed using the 3D Slicer software, a free and open-source application for medical image processing. In the realm of clinical research, 3D Slicer functions akin to a radiologic workstation, facilitating comprehensive visualizations and advanced features such as automated segmentation and registration for diverse application domains. The 3D Slicer software serves as a comprehensive tool for the qualitative and quantitative exploration of multimodal medical imaging data [30]. In the present study, this application was employed to conduct highly precise radiomorphometric measurements on pantomographic images of the enrolled subjects.

Furthermore, the morphological classification of the cortical bone of the mandible was performed on pantomographic radiographs. The mandibular cortical index (MCI) was utilized for evaluating morphological changes in the mandibular compact bone. Cortical shapes were analyzed through the observation of the mandible distal to the bilateral mental foramina, and classified into one of the three following groups:

C1—Inner border of the compact bone exhibits smooth uniformity on both sides;
C2—Inner border of the compact bone displays crescent-shaped defects (lacunar resorption) on one or both sides, featuring mild to moderate cortex erosion;
C3—Compact bone displays evident porosity [31].

Lateral images of the temporomandibular joints (TMJs) were captured as per the protocol of the GXDP-300 (Gendex Dental Systems, Hatfield, PA 19440, USA) digital radiographic apparatus manufacturer. The acquisition of TMJ lateral radiographs aimed to assess the bony structures of the condyle. Conventional radiography proves valuable in depicting degenerative joint disease in advanced stages and allows for the evaluation of condylar positioning within the TMJs [32].

### 2.4. Statistical Analysis

In evaluating the intra-class measurement consistency of MCW in an interval scale, the Intraclass Correlation Coefficient (ICC) was employed. The ICC (2.1) variant, following the conventions outlined by Shrout and Fleiss, was utilized. Koo and Li propose the subsequent interpretation for ICC values—below 0.50: poor, between 0.50 and 0.75: moderate, between 0.75 and 0.90: good, above 0.90: excellent [33].

Statistical significance of differences between two quantitative groups was assessed using the Mann–Whitney U test. Fisher’s exact test was applied for categorical data (2 × 2 tables). Fisher’s exact test was preferred due to the small sample size; moreover, Fisher’s exact test is superior to the chi-squared test in terms of accuracy. Additionally, the odds ratio (OR) was computed along with 95% confidence intervals. For statistical significance of differences across more than two categorical groups, the Freeman–Halton extension of Fisher’s exact test (Fisher–Freeman–Halton test) was employed [34].

For statistical analyses necessitating conformance of the variable distribution to a normal (symmetric) distribution, verification was executed via the Shapiro–Wilk test. Non-parametric tests were only employed in scenarios where parametric testing was unfeasible. A significance level of *p* < 0.05 was adopted for all analyses [35].

As an ancillary statistical method for the hypothesis validation and determination of predictive power, logistic regression (single-factor regression between the independent variable and the dependent dichotomous variable) was employed. The logistic regression model represents a generalized linear model, employing the logit function as the linking mechanism. Classification quality was assessed by calculating the Area Under the ROC Curve (AUC), providing an aggregated measure of performance across all possible classification thresholds, and delivering a comprehensive description of sensitivity and specificity [35,36].

For the assessment of the relationship level (range [−1, 1]) between variables with a normal distribution, the Pearson linear correlation coefficient, or the point-biserial correlation coefficient, was utilized for continuous and nominal (dichotomous) variables. For variables failing to meet the normal distribution requirement, the Spearman correlation coefficient (a non-parametric correlation based on ranks) was employed. Evaluation was directed toward the absolute value of the correlation, which describes its strength and direction. Results for continuous variables were presented in tables, including sample sizes (N), mean, standard deviation, median, minimum, and maximum [35]. All computations were conducted using computational methodologies through the R language (packages: irr and PerformanceAnalytics, version: 4.1) and Python (packages: pandas, sklearn, scipy, and statsmodels, version: 3.10).

## 3. Results

### 3.1. Patients’ Group

In the initial study, an assessment of oral health status was conducted in both groups, with the results being expressed in the form of the DMFT index. The assessment of the presence of plaque and calculus was recorded in terms of OHI-S scores. The presented results depicted the oral health status in the study and control groups. Table 3 presents the baseline oral health status, evaluated based on WHO criteria utilizing the DMFT and OHI-S indices.

**Figure 3:**
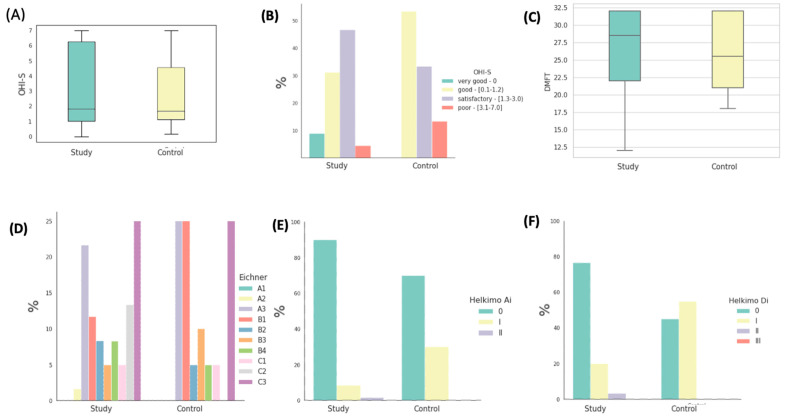
Graphical representation of statistical analysis. (**A**) Distribution of OHI-S index values in the investigated groups. (**B**) The proportions of patients within the studied groups classified into four interpretative intervals of the OHI-S index. (**C**) The distribution of DMFT (Decayed, Missing, Filled Teeth) index values in the studied groups. (**D**) Percentages of patients in the studied groups classified into interpretative intervals of Eichner’s classes. (**D**) Percentage distribution of patients within the studied groups categorized into three interpretative intervals of the Helkimo Ai index. (**E**) Percentage distribution of patients within the studied groups categorized into three interpretative intervals of the Helkimo Ai index. (**F**) Percentages of patients in the studied groups classified into four interpretative intervals of the Helkimo Di index.

In the investigated cohort comprising all patients, the mean Oral Hygiene Index—Simplified (OHI-S) manifested an average of 1.53 ± 1.09. Within the subset of patients allocated to the study group, the mean OHI-S registered at 1.51 ± 1.12, whereas in the control group, it recorded 1.56 ± 1.01. Among patients afflicted with oral malignancies, the most prevalent subgroup was constituted by individuals exhibiting satisfactory oral hygiene practices, accounting for 48.89% of the cohort. Their mean OHI-S score was 1.82 ± 0.42. In contrast, within the control group, a majority of 53.33% exhibited commendable oral hygiene practices, yielding a mean OHI-S score of 0.85 ± 0.35. It should be noted that the OHI-S index calculations were restricted to patients possessing dentition. Conducting a statistical analysis, we failed to detect any statistically significant distinctions pertaining to OHI-S index values between the two compared groups (*p* = 0.500) (Figure 3A,B). The mean DMFT (Decayed, Missing, Filled Teeth) index value in the study group (Figure 3C) was 26.35 ± 6.22, with the mean number of missing teeth (M) averaging 18.91. In the control group, the mean value of this index was 25.60 ± 4.97, with a mean number of missing teeth at 15.94. The statistical analysis revealed no significant differences in DMFT index values between the study group and the control group (*p* = 0.262), nor in the number of missing teeth within this index (*p* = 0.335) between the groups.

In the study group, encompassing patients with fully edentulous arches in occlusal contact, no instances of dental deficiencies were observed. However, within this cohort, 23.33% of patients exhibited support in all lateral zones (A2 + A3), 33.33% presented with a complete absence of preserved support zones, 18.33% displayed a lack of mutual contacts between opposing teeth (C1 + C2), and 25% were characterized by complete edentulism in both maxillary and mandibular arches.

Within the control group, similarly comprising patients with full dental arches in occlusal contact, no instances of dental deficiencies were noted. Nevertheless, 25% of these patients exhibited support in all lateral zones (A2 + A3), while the predominant subgroup consisted of patients with a complete absence of preserved support zones, accounting for 45%. Furthermore, 5% of subjects exhibited a lack of mutual contacts between opposing teeth (C1 + C2), and 25% were characterized by complete edentulism. The distribution of the percentages of patients categorized based on Eichner’s classification within the respective groups is visually represented in Figure 3D.

Following the application of the Helkimo anamnestic index in the study group, a mere five patients acknowledged experiencing mild discomfort, such as acoustic phenomena or other mild symptoms (classified as Ai-I), comprising a mere 8.33% of the study cohort. Only one patient reported pain as the predominant symptom (Ai-II), accounting for 1.67% of the afflicted individuals. The remaining 90% of the study group were categorized into the Ai-0 group, as they did not report any discomfort or symptoms.

In the control group, 14 patients were devoid of any reported complaints related to the masticatory system, constituting 70% of the entire control cohort. Six patients reported mild acoustic symptoms and muscle fatigue, representing 30% of the control group. None of the patients reported advanced symptoms. The distribution of the percentages of patients classified into three interpretative categories of the Helkimo Ai index is graphically depicted in Figure 3E.

In the study group, clinical assessment revealed a lack of clinical symptoms indicating Di-0 dysfunction in 46 participants (76.67%). Di-I dysfunction, indicative of mild dysfunction, was diagnosed in 12 patients, comprising a total of 20% of the study population. Symptoms described by Di-II, classified as moderate dysfunction, were observed in two individuals, accounting for 3.33%. Notably, none of the patients were diagnosed with Di-III, characterized as severe dysfunction. Conversely, in the control group, clinical examination did not identify any signs of temporomandibular system dysfunction in 45% (nine individuals) of the participants. Among the remaining 55% (11 patients) of the control group, mild dysfunction was detected, with no patients presenting with moderate (Di-II) or severe (Di-III) dysfunction. Statistically significant differences in the degree of dysfunction between the two groups were observed (*p* = 0.01). Figure 3F illustrates the distribution of the percentages of patients classified into four interpretative groups of the Helkimo Di index. It is noteworthy that a higher prevalence of severe dysfunction can be observed in the control group.

The initial occlusal conditions using Eichner’s classification and the degree of temporomandibular joint dysfunction assessed using the Helkimo index in the first examination are presented in Table 4.

Table 5 presents the initial level of gingival inflammation using the mSBI index (in percentages), as well as the advancement in periodontal structure destruction by measuring pocket depth (PD) (in millimeters) and clinical attachment loss (CAL) (in millimeters).

**Figure 4:**
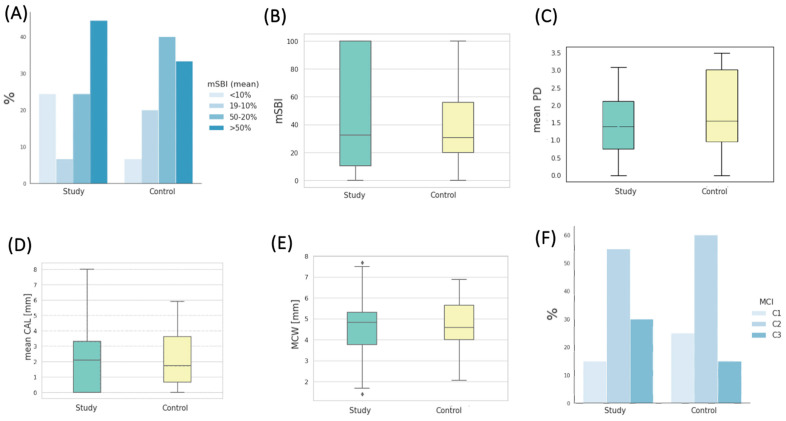
(**A**) Percentages of patients in the studied groups classified into four interpretative intervals of the mSBI index. (**B**) Distribution of mSBI values in the studied groups. (**C**) Distribution of the mean PD (pocket depth) values in the studied groups. (**D**) Distribution of the mean CAL (clinical attachment loss) values in the studied groups. (**E**) Distribution of MCW (mandibular cortical width) values in the studied groups. (**F**) Percentages of patients in the studied groups classified into three interpretative intervals of the MCI (mandibular cortical index).

The level of gingival inflammation, as assessed by the mSBI (modified Sulcus Bleeding Index), was notably elevated within both patient cohorts. In the study group, the largest contingent, accounting for 44.44% of patients, exhibited mSBI values exceeding 50%, with a remarkably high mean mSBI value of 93.14 ± 13.37%. In contrast, among patients in the control group, the most prevalent subgroup, comprising 40%, displayed mSBI values ranging from 50% to 20%, with an average mSBI value of 32.88 ± 9.98%. The distribution of percentages across the four interpretative ranges of mSBI, illustrated in Figure 4A, did not demonstrate any statistically significant differences between the study groups as a whole. The mean mSBI values for both groups are presented in Figure 4B.

Parameters characterizing the degree of periodontal tissue destruction are also presented in Table 5. The mean pocket depth (PD in mm) in the study group was 1.82 ± 0.63, whereas in the control group, it measured 2.32 ± 0.85 (depicted in Figure 4C). Notably, the difference in pocket depth between the study groups exhibited statistical significance (*p* = 0.017). The mean clinical attachment loss (CAL in mm) in the study group was 2.91 ± 1.48, slightly higher (without statistical significance) than in the control group, where it measured 2.78 ± 1.59, as depicted in Figure 4D. The mean measurement of the width of the mandibular cortical bone (MCW in mm) in the study group was 4.60 ± 1.31, while in the control group, it was 4.58 ± 1.38. The statistical analysis did not reveal significant differences in MCW values between the groups at the baseline (Figure 4E). A Shapiro–Wilk test was conducted for the study sample, indicating no grounds to reject the hypothesis of the normal distribution of MCW (*p* = 0.61). The MCW measurements were performed by three dentists. The Intraclass Correlation Coefficient (ICC) test was applied to assess the measurement reliability among different assessors. An ICC close to 1 signifies a high level of agreement among the assessors. In this study, the ICC was 0.968, with a 95% CI of 0.859 < ICC < 0.996, and *p* < 0.001, indicating a high level of agreement among the obtained results among the assessors. On the other hand, the mean PMI (panoramic mandibular index) value in the study group was 0.3156, while in the control group, it was 0.3361. These values did not differ significantly between the groups (*p* = 0.367).

Regarding the mandibular cortical index (MCI), the majority of patients in the study group had C2 (55%) and C3 (30%), respectively. In the control group, the most common MCI was C2, observed in 60% of patients, followed by C1, present in 25% of patients. The distribution of percentages across the three interpretative ranges of MCI is depicted in Figure 4F. Table 6 contains measurements taken during the initial examination using panoramic radiography, represented by the MCW measurement and radiomorphometric indices PMI and MCI.

### 3.2. Prevalence of Risk Factors for Avascular Necrosis of the Jaw in the Investigated Cohorts

Maintaining proper oral hygiene plays a pivotal role as a risk factor for avascular necrosis of the jaw among patients undergoing bisphosphonate therapy. In the study cohort, 56.66% (34 patients) reported engaging in tooth or denture brushing routines at least twice daily (two and three times daily). This percentage was statistically lower compared to the control group, where 80% (16 patients) followed similar oral hygiene practices. Conversely, 11.67% (seven patients) of individuals in the study group admitted to not engaging in any tooth or denture brushing, while in the control group, only one patient (5%) reported a similar behavior.

Furthermore, in the study group, a mere 31.67% (19 patients) employed additional oral hygiene measures such as mouthwashes, dental floss, irrigators, and interdental brushes. The majority of patients resorted to mouthwashes, with 17 patients endorsing their use. Additionally, two patients combined dental floss with mouthwash, one patient solely employed dental floss, and two patients made use of an irrigator. In contrast, the control group exhibited a significantly higher prevalence of these supplementary oral hygiene practices at 70% (14 patients).

The use of glucocorticoids, recognized as one of the risk factors in the existing literature, was reported by 8.33% of the study group (five patients). Conversely, neither the remaining study group nor the entire control group was under the influence of medications from this class.

Amongst patients receiving bisphosphonate treatment, 11.67% (seven individuals) admitted to being smokers in the survey. Conversely, in the control group, 20% (four patients) disclosed their smoking status.

Regarding alcohol consumption, 40% (24 patients) in the study group acknowledged consuming alcoholic beverages. In contrast, within the control group, 65% (13 individuals) reported alcohol consumption. Notably, no statistically significant discrepancies emerged between these two groups in relation to this risk factor.

In terms of prosthetic dentistry, 48.33% (29 patients) of the study group possessed removable dentures, while 18.33% (11 patients) had fixed dentures, and 33.33% (20 patients) did not have any form of denture. Conversely, in the control group, 50% (10 patients) utilized removable dentures, with 40% (8 individuals) having fixed dentures, and 10% (2 patients) being completely edentulous. The variations in the prevalence of denture usage between the two groups did not exhibit statistical significance. A detailed overview of the interplay among these risk factors in both study cohorts is presented in Table 7.

### 3.3. Changes in Selected Parameters in the Study and Control Groups

In order to show the results of all the indicators studied after 6 months in both study groups, we compiled them in tabular form (Table 8).

The comparison of changes in selected parameters in the study and control groups in two clinical and radiological studies is presented in Table 9.

**Figure 5:**
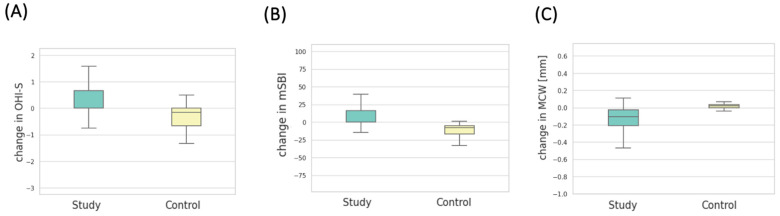
(**A**) Distribution of OHI-S change values in the study groups. (**B**) Distribution of mSBI change values in the study groups. (**C**) Distribution of MCW change values in the study groups.

A statistically significant increase in the OHI-S index was observed among patients in the experimental group compared to the control group (*p* < 0.001). In the experimental group, the mean OHI-S score at baseline was 1.51 ± 1.12, while after 6 months, it was 1.89 ± 0.98. In the control group, the mean OHI-S value was 1.56 ± 1.01 at the beginning and 0.98 ± 0.48 after 6 months. The change in OHI-S in both groups after 6 months is depicted in Figure 5.

There were no statistically significant differences observed in the assessment of anamnestic (*p* = 0.420) and clinical (*p* = 0.339) Helkimo dysfunction indices in both groups after a 6-month period.

Among patients who initiated bisphosphonate treatment, no statistically significant increase in CAL (*p* = 0.286) was noted in comparison to patients in the control group during the six-month observation period. However, a noticeable increasing trend in this index was observed in the experimental group compared to the control group. In the experimental group, the mean CAL was initially 2.91 ± 1.48, and after 6 months, it was 2.98 ± 1.49. In the control group, the mean CAL value was 2.78 ± 1.59 at the start and 2.84 ± 1.59 after 6 months.

There was no statistically significant difference in the change in the mean PD value between the study groups in the two studies (*p* = 0.400). In the experimental group, the mean PD (mm) before bisphosphonate therapy was 1.82 ± 0.63, and in the second study, it was 1.87 ± 0.65. In the control group, the mean PD was 2.32 ± 0.85 in the first study and 2.35 ± 0.83 after six months.

Patients after 6 months of bisphosphonate treatment had a statistically significant increase in the mSBI (%) index compared to patients in the control group (*p* = 0.001). In the first study, the mean mSBI in the experimental group was 49.12 ± 42.00, and in the second study, it was 59.69 ± 39.35. In the control group, the mean mSBI value was 40.25 ± 28.75 in the first study and 25.98 ± 19.74 in the second. The change in the mSBI index over 6 months in both groups is depicted in Figure 5B.

In the measurements taken from panoramic radiographic images, a statistically significant increase in MCW values was observed in patients from the experimental group after 6 months of therapy with the drug, in comparison to the measurements from radiographic images of patients in the control group (*p* = 0.001). The average MCW (mm) in the experimental group prior to medication initiation was 4.60 ± 1.31, and after 6 months, it was 4.73 ± 1.34. The same measurements in the control group were 4.58 ± 1.38 in the initial examination and 4.56 ± 1.38 in the examination after 6 months. The change in MCW measurement over the course of 6 months in both groups is depicted in Figure 5C.

Furthermore, the h (mm) measurement was assessed on radiographic images in both study groups. It was observed that the h dimension did not significantly change statistically in the six-month time frame in either group.

Change in h in the experimental group: Mann–Whitney U test, *p* = 0.48.

Change in h in the control group: Mann–Whitney U test, *p* = 0.45.

Based on the aforementioned radiomorphometric measurements, the PMI index was calculated, and its change over time was statistically significantly higher in the experimental group than in the control group. In the experimental group, in the first study, the average PMI value was 0.3156 ± 0.1035, and in the second study, it was 0.3245 ± 0.1050. Meanwhile, the average PMI in the first study was 0.3361 ± 0.1223, and in the second study, it was 0.3338 ± 0.1218.

### 3.4. Correlations between Selected Parameters

#### 3.4.1. Correlation between Patient Age and Tooth Brushing Frequency in Respective Groups

**Figure 6:**
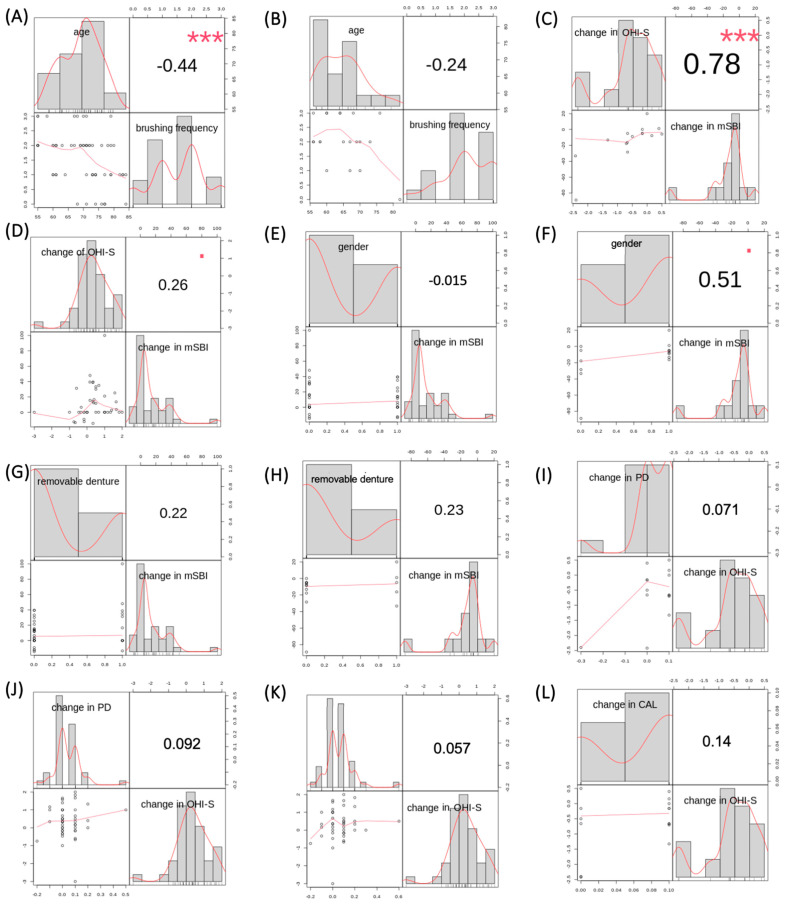
(**A**) Correlation results between tooth brushing frequency and age in the experimental group. (**B**) Correlation results between tooth brushing frequency and age in the control group. (**C**) Correlation results between changes in OHI-S and mSBI in the control group. (**D**) Correlation results between changes in OHI-S and mSBI in the experimental group. (**E**) Correlation results between gender and changes in mSBI in the experimental group. (**F**) Correlation results between gender and changes in mSBI in the control group. (**G**) Correlation results between the usage of removable dentures and changes in mSBI in the experimental group. (**H**) Correlation results between the usage of removable dentures and changes in mSBI in the control group. (**I**) Correlation results between changes in PD and OHI-S in the control group. (**J**) Correlation results between changes in PD and OHI-S in the experimental group. (**K**) Correlation results between changes in CAL and OHI-S in the experimental group. (**L**) Correlation results between changes in CAL and OHI-S in the control group. Results with asterisks refer to Fisher’s exact test.

An inverse correlation was observed in the experimental group, indicating that as patients in the experimental group grew older, their tooth brushing frequency decreased significantly (*p* < 0.001; corr = −0.44) (Figure 6A). However, there was no correlation between tooth brushing frequency and age in the control group (*p* = 0.31, corr = −0.24) (Figure 6B).

#### 3.4.2. Correlation between Changes in OHI-S and mSBI Indices in Respective Groups

A comparison of the changes in the mSBI index in correlation with changes in the OHI-S index was performed in both groups—experimental and control. A decrease in the OHI-S index in the control group correlated with a decrease in the mean mSBI value in the control group (*p* < 0.001, corr = 0.78) (Figure 6C). In the experimental group, there was no correlation between an increase in the OHI-S index and an increase in the mean mSBI value (*p* = 0.09, corr = 0.26), as shown in Figure 6D.

#### 3.4.3. Correlation between Gender and Changes in mSBI Values in Respective Groups

Comparing changes in the mSBI index with gender in each group, it was found that there was no correlation between changes in the mSBI index over a 6-month period and gender in either of the study groups: experimental group—*p* = 0.92, corr = −0.015 (Figure 6E), control group—*p* = 0.05, corr = 0.51 (Figure 6F).

#### 3.4.4. Correlation between Removable Denture Usage and Changes in mSBI Values in Respective Groups

Comparing changes in the mSBI index with the usage of removable dentures in each group, it was found that there was no correlation between changes in the mSBI index over a 6-month period and the usage of removable dentures in either of the study groups: experimental group—*p* = 0.14, corr = 0.22 (Figure 6G), control group—*p* = 0.41, corr = 0.23 (Figure 6H).

#### 3.4.5. Correlation between Changes in Average PD Measurement and Changes in OHI-S Values in Respective Groups

A comparison of changes in average PD measurement in correlation with changes in the OHI-S index was conducted in both groups—experimental and control. A decrease in the OHI-S index in the control group did not correlate with a decrease in the mean PD value in the control group (*p* = 0.80, corr = 0.07). Figure 6I illustrates this relationship. In the experimental group, there was also no correlation between an increase in the OHI-S index and an increase in the mean PD value (*p* = 0.55, corr = 0.09), as shown in Figure 6J.

#### 3.4.6. Correlation between Changes in Average CAL Measurement and Changes in OHI-S Values in Respective Groups

A comparison of changes in average CAL measurement in correlation with changes in the OHI-S index was performed in both groups—experimental and control. The results are presented in Figure 6K,L. There was no correlation between an increase in the OHI-S index and an increase in the mean CAL value in both the experimental group (*p* = 0.71; corr = 0.06) and the control group (*p* = 0.61; corr = 0.14), respectively.

## 4. Discussion

Bisphosphonate-induced osteonecrosis of the jaws (BIONJ) was first reported by Marx and Stern in 2002 [37,38,39]. BIONJ negatively impacts the quality of life of patients in relation to the function of the stomatognathic system—impairing proper chewing, hindering speech, causing significant discomfort and occasional pain, impeding standard dental treatment, and requiring specialized care [40]. Despite numerous observations and scientific data regarding the pathophysiology, ultimate treatment strategies of BIONJ are limited. Therefore, it is imperative to elucidate the precise mechanisms of its development and establish BIONJ treatment strategies [41]. Thus, this study focused on the changes that occurred in patients taking bisphosphonates for 6 months and the dynamics of these changes in generally healthy patients who did not take this medication.

Authors investigating the risk factors that may influence the occurrence of bisphosphonate-related osteonecrosis of the jaw (BRONJ) are in agreement that one of them is poor oral hygiene [42,43,44]. In the conducted study, the majority of patients with cancer in the initial examination had satisfactory oral hygiene, 48.89%, whereas in the control group, the majority of patients had good oral hygiene—53.33%. In a subsequent study after 6 months, the proportion of patients with satisfactory oral hygiene increased to 60%, and in the control group with good oral hygiene, it increased to 73.33%. As a result, the deterioration of oral hygiene was statistically significant in the study group compared to the control group during the study.

Scientific research emphasizes the importance of oral health in patients already diagnosed with bisphosphonate-related osteonecrosis (BRONJ). In the conducted study, the DMFT index did not differ statistically between both groups, but in both groups, it was at a high level, exceeding 20. The average DMFT in the study group was 26.35, and the average in the control group was 25.60. In the study by Krimmel et al., the DMFT index was employed as an indirect indicator of oral and dental health, with a value assessed before commencing BP treatment [45]. During this period, patients had a DMFT index of 20.5 ± 4.2. A significant difference was observed in the time of BRONJ onset between patients with a DMFT index below and above 20. Patients with a DMFT index ≤ 20 developed BRONJ after 39.7 ± 1.1 months, while patients with a DMFT index > 20 exhibited symptoms of BRONJ after only 14.4 ± 2.8 months (*p* < 0.001). An observational study by Wazzan also disclosed that patients with 15 or more missing teeth are at high risk of developing MRONJ (medication-related osteonecrosis of the jaw) [46]. In our own study, the average number of extracted teeth in the study group was 19.09, and in the control group, it was 15.94. They did not differ statistically, but these figures exceeded the number of 15 reported in other studies as a risk factor for medication-related osteonecrosis in patients taking bisphosphonates.

In the study by Caldas et al., it was shown that oral infections seemed to play a significant role in the development of bone necrosis [47]. Additionally, advanced stages of MRONJ were associated with higher DMFT indices. In the study by Kos et al., patients taking bisphosphonates and diagnosed with BRONJ had poorer oral hygiene, more dental caries complications, and worse periodontal health compared to individuals without BRONJ [48]. Indeed, the analysis of our survey results in this study suggests significant shortcomings in comprehensive dental prophylaxis among patients with cancer. Only 56.6% of patients in the study group brushed their teeth or dentures 2–3 times a day, while in the control group, this number was 80%. Importantly, there was a correlation where older patients in the study group brushed their teeth less frequently (*p* < 0.001). However, there was no correlation between brushing frequency and age in the control group (*p* = 0.31). Additionally, the percentage of patients in the study group using additional oral hygiene measures was notably low at 31.67%, whereas in the control group, it was a satisfactory 70% of patients.

Patients on intravenous bisphosphonates often have advanced cancer, struggle to maintain regular oral hygiene, and face frequent hospitalization. Their vulnerability to oral infections stems from factors such as frailty, systemic diseases, compromised immunity, and multifactorial causes [49]. Consequently, the buildup of dental plaque and calculus near the gum line can lead to periodontal attachment degradation, causing inflammation and eventually alveolar bone involvement. The risk of jawbone necrosis in patients with cancer is substantially higher than in healthy individuals, up to four times greater. Interestingly, Ripamonti et al. have shown that improving oral hygiene empirically reduces the incidence of BRONJ in patients with multiple myeloma and metastatic cancer [50]. In this context, patients receiving bisphosphonate treatment should be provided with dental care to manage oral infections and decrease the risk of complications like MRONJ. Preventative measures include a comprehensive oral examination, addressing dental issues before starting bisphosphonate therapy, antibiotic treatment, avoiding invasive dental procedures for patients on BP treatment, and identifying risk factors [38,51]. As mentioned earlier, the findings of Ripamonti et al. support published clinical guidelines, highlighting a considerable reduction in ONJ frequency when patients receive appropriate dental prophylaxis in collaboration with oncologists [50].

Both advanced age and chemotherapy-induced immunosuppression are established risk factors for periodontal diseases. Marx et al. highlighted that a striking 84% of patients with BRONJ had pre-existing periodontal disease, including nearly a third with advanced cases [39]. A comprehensive review by Hoff and colleagues similarly underlined a substantial prevalence (41%) of periodontal inflammation among patients with BRONJ [52]. In our initial study, the mean pocket depth (PD) exhibited a statistically significant difference between both the study and control groups, measuring 1.82 mm in the study group and 2.32 mm in the control group. However, clinical attachment loss (CAL) did not significantly differ between the groups, with measurements of 2.91 mm in the study group and 2.78 mm in the control group. Notably, both groups displayed elevated gum inflammation levels, as indicated by the mSBI index. In the study group, 44.44% of patients had an mSBI exceeding 50%, with an average mSBI of 49.12%. In the control group, 40% of patients had an mSBI ranging from 50% to 20%, with an overall average mSBI of 40.25%. Following 6 months of observation, changes in PD and CAL did not exhibit statistically significant differences between the two groups. However, a substantial and statistically significant deterioration in gum inflammation was observed in the study group compared to the control group (*p* = 0.001).

It is important to acknowledge the influence of local factors, notably inadequate oral hygiene, on periodontal health. Grgić et al.’s study highlighted that patients with osteoporosis taking oral bisphosphonates demonstrated higher values in the gingival index, plaque index, and bleeding [53]. In our study, the decrease in the OHI-S index in the control group did not correlate with a decrease in the mean PD in the same group (*p* = 0.80). Similarly, in the study group, there was no correlation between an increase in the OHI-S index and an increase in the mean PD (*p* = 0.55). With regards to CAL measurements in both the study and control groups, there was no correlation between an increase in the OHI-S index and an increase in the mean CAL (*p* = 0.71 and *p* = 0.61, respectively). Notably, the worsening of gum inflammation in the study group did not significantly correlate with a deterioration in oral hygiene (*p* = 0.09). In contrast, in the control group, there was a significant correlation between the improvement in gum inflammation and the enhancement in oral hygiene (*p* < 0.001). It is reasonable to propose that intravenously administered bisphosphonates in the study group may have contributed to the statistically significant increase in mSBI values, consequently resulting in the exacerbation of gum inflammation. Kos’s study also observed significantly poorer gum bleeding parameters in patients with BRONJ when compared to individuals without BRONJ symptoms [48]. It is worth noting that bisphosphonates can exert their effects not only on bone, which is their primary target tissue, but also on adjacent soft tissues.

Bisphosphonates can lead to increased mineralization in the coronoid process of the mandible. They may also inhibit cartilage tissue resorption, as demonstrated by Kim et al., with alendronate reducing articular cartilage degradation. Consequently, bisphosphonates might improve bone mineralization and inhibit angiogenesis, thus contributing to temporomandibular joint (TMJ) inflammation treatment [54]. In our study, a functional analysis of the stomatognathic system using the anamnestic and clinical components of the Helkimo dysfunction index showed significant differences between the study and control groups initially. However, predicting group membership based on these indices was not possible. After six months, no significant changes were observed in either group. Radiographic imaging did not yield significant results in either group, and the lack of a noticeable effect of bisphosphonates on TMJ dysfunction in the study group may be due to the relatively short observation period.

Importantly, our study found that 90% of the study group and 70% of the control group were symptom-free in terms of stomatognathic system issues according to the anamnestic Helkimo index Ai-0. Additionally, 76.67% of study group patients exhibited Helkimo clinical dysfunction index Di-0, whereas 55% of control group patients had Di-I. After a 6-month follow-up, no statistically significant changes were observed in either group for the anamnestic (*p* = 0.420) and clinical dysfunction indices (*p* = 0.339). Radiological examinations (X-rays) did not yield significant results in either group. Regarding the risk factors for ONJ in patients undergoing intravenous bisphosphonate therapy, smoking was found to be a probable risk factor, as shown by Wessel et al. [55]. Jadu et al. identified glucocorticosteroid use as a statistically significant risk factor. In this study, no significant differences were observed in smoking habits (*p* = 0.450) or alcohol consumption (*p* = 0.07) between the study and control groups [56]. Only 11.67% of study group patients and 20% of control group patients were regular smokers. Similarly, 40% of study group patients and 65% of control group patients confirmed alcohol consumption. Regarding glucocorticosteroid use, only 8.33% of study group patients and none in the control group had used these medications in the last three months (*p* = 0.32).

Denture-wearing patients with cancer receiving intravenous bisphosphonates had an increased risk of ONJ due to chronic oral tissue irritation. Denture maintenance during intravenous therapy is recommended to prevent oral mucosal damage. An analysis of occlusal conditions was performed using the Eichner classification. Missing teeth affected the way forces were transferred between dental arches during chewing. Patients in the study group required more prosthodontic treatment, primarily complete or partial dentures, compared to the control group. After therapy, the study group patients with removable dentures had fewer issues, whereas control group patients experienced problems and required adjustments or metal clasps.

Significantly, panoramic radiographs showed an increase in mandibular cortical width (MCW) in the study group after 6 months of medication therapy compared to the control group (*p* < 0.001). Our study found that the h-dimension, the distance from the mental foramen to the lower mandibular bone border, did not significantly change over six months in both groups (*p* = 0.48 in the study group, *p* = 0.45 in the control group). The panoramic mandibular index (PMI) showed no initial significant difference between the groups, but was significantly higher in the study group after 6 months. Torres et al. observed a significant MCW difference in three groups (*p* < 0.001), with higher average MCW in both BRONJ and non-BRONJ groups compared to controls (*p* < 0.0001 and *p* = 0.006, respectively) [57]. No significant differences were noted when comparing zoledronic acid users to other bisphosphonate users (*p* = 0.37). In Kubo et al.’s study, MCW was not a BRONJ indicator. MCW was larger on the affected side in the ONJ+ group but smaller in the ONJ− group compared to other groups. They suggested the C2 MCI class as a potential BRONJ predictor [58]. Our study found the highest C2 class frequency at the initial examination, with notable changes observed in the study group after 6 months.

Ozcan et al. reported a high frequency of C1-class MCI in BRONJ sites on panoramic radiographs compared to the control group, predominantly the C2 class [59]. Ogura et al., using PanoSCOPE, also found a majority of C1-class MCI in patients with MRONJ and bone metastases [60]. Other researchers have noted MCW variations in patients with BRONJ symptoms compared to those on bisphosphonate therapy without necrosis symptoms. Our study observed an MCW increase dependent on medication intake over 6 months. None of the patients in our study group showed jawbone necrosis symptoms within 6 months of receiving intravenous bisphosphonates, likely due to the short observation period and initial dental screening excluding major dental risk factors.

In this study, we explored the impact of bisphosphonate therapy on oral health among patients with breast and prostate cancer and bone metastases. Despite our comprehensive approach, we acknowledge limitations such as the study’s sample size and the observational design, which may limit generalizability. Additionally, the quantification of BRONJ symptoms and long-term effects on the stomatognathic system warrant further investigation. Future research should aim to include larger, diverse cohorts and longitudinal data to fully understand bisphosphonates’ implications on oral health. These insights will guide the development of more effective dental care protocols for this patient population.

## 5. Conclusions

In conclusion, patients with breast and prostate cancer exhibited suboptimal oral hygiene levels before treatment, which further deteriorated after six months of medication use, primarily due to infrequent brushing and limited additional oral hygiene measures. The cancer group experienced more significant gum inflammation than the control group after six months, potentially linked to bisphosphonate intake. Interestingly, gum inflammation in the cancer group was not associated with declining oral hygiene, suggesting a potential influence of intravenous bisphosphonates. However, no significant changes were observed in periodontal structure between the groups, likely due to medication ineffectiveness or the relatively short observation period. Both groups had a notable number of missing teeth, which could increase the risk of jawbone necrosis in patients with cancer taking bisphosphonates. Removable dentures did not significantly impact stomatognathic system changes. Changes in the prosthetic foundation were minimal after six months, requiring more extended clinical observation. Patients on bisphosphonates exhibited increased measurements on radiographs, indicating bisphosphonate effects on bones and stomatognathic system changes. Implementing radiological dental diagnostics in patient monitoring before and during bisphosphonate therapy is justified. Assessing the stomatognathic system before and during intravenous bisphosphonate therapy in patients with metastatic bone cancer offers several advantages, such as an early detection of oral changes preceding jawbone necrosis and appropriate treatment. Involving dentists and dental hygienists in multidisciplinary patient care for those at risk of bisphosphonate-related jawbone necrosis is essential. Additionally, it is crucial to recognize the potential risks associated with bisphosphonate therapy, especially in the context of bisphosphonate-related osteonecrosis of the jaw (BRONJ). Poor oral hygiene was identified as a risk factor in this study, and dental prophylaxis should be an integral part of the care for patients undergoing bisphosphonate treatment. The study’s findings underscore the importance of maintaining oral health in patients receiving bisphosphonates to reduce the risk of complications like MRONJ. This study has shed light on the oral health dynamics in patients with cancer undergoing bisphosphonate therapy and emphasizes the need for a multidisciplinary approach to their care, with a focus on oral health management and close monitoring.

## Figures and Tables

**Table 1:** Compilation of Age-Related Data in Investigated Groups.

Quantitative Variables		Group		Mann–Whitney U Test
		Test Group	Control Group	
Age	N (%)	60 (100%)	20 (100%)	*p* = 0.009
	Mean	69.6	64.9	
	SD	8.3	7.2	
	Median	71.0	64.5	
	Range	55.0–84.0	56.0–82.0	

**Table 2:** Compilation of gender-related data in the studied groups.

Categorical Variables			Group		Fisher Exact Test
			Tested	Control	
Gender	F	N (%)	22 (36.67%)	13 (65.00%)	*p* = 0.04 OR: 0.32 (95% CI: 0.09–1.01)
	M	N (%)	38 (63.33%)	7 (35.00%)	

**Table 3:** Results of the statistical analysis of DMFT and OHI-S indices for the studied groups in the first examination.

Quantitative Variables			Group		Mann–Whitney U Test
			Study	Control	
DMFT		N (%)	60 (100%)	20 (100%)	*p* = 0.262
		Mean	26.35	25.60	
		Std. Deviation	6.22	4.97	
		Median	28.50	25.50	
		Range	12.00–32.00	18.00–32.00	
M—number of extracted teeth		N (%)	60 (100%)	20 (100%)	*p* = 0.335
		Mean	18.91	15.94	
		Std. Deviation	10.39	10.99	
		Median	17.00	11.00	
		Range	3.00–32.00	5.00–32.00	
OHI-S	0 (excellent)	N (%)	4 (8.89%)	0 (0%)	*p* = 0.500
		Mean	0.00	-	
		Std. Deviation	0.00	-	
	0.1–1.2 (good)	N (%)	14 (31.11%)	8 (53.33%)	
		Mean	0.71	0.85	
		Std. Deviation	0.33	0.35	
OHI-S	1.3–3.0 (satisfactory)	N (%)	22 (48.89%)	5 (33.33%)	*p* = 0.500
		Mean	1.82	1.89	
		Std. Deviation	0.42	0.38	
	3.1–6.0 (poor)	N (%)	5 (11.11%)	2 (13.33%)	
		Mean	3.67	3.58	
		Std. Deviation	1.31	0.25	
OHI-S mean		N (%)	45 (100%)	15 (100%)	
		Mean	1.51	1.56	
		Std. Deviation	1.12	1.01	
		Median	1.33	1.17	
		Range	0.00–6.00	0.17–3.75	

**Table 4:** Results of statistical analysis of Eichner’s classes and Helkimo indices for the studied groups in the initial examination.

Qualitative Variables			Group		Fisher’s Exact Test
			Study	Control	
Eichner	A1	N (%)	0 (0.00%)	0 (0.00%)	*p* = 0.65
	A2	N (%)	1 (1.67%)	0 (0.00%)	
	A3	N (%)	13 (21.67%)	5 (25.00%)	
	B1	N (%)	7 (11.67%)	5 (25.00%)	
	B2	N (%)	5 (8.33%)	1 (5.00%)	
	B3	N (%)	3 (5.00%)	2 (10.00%)	
	B4	N (%)	5 (8.33%)	1 (5.00%)	
Eichner	C1	N (%)	3 (5.00%)	1 (5.00%)	*p* = 0.65
	C2	N (%)	8 (13.33%)	0 (0.00%)	
	C3	N (%)	15 (25.00%)	5 (25.00%)	
Eichner	A	N (%)	14 (23.33%)	5 (25.00%)	*p* = 0.57
	B	N (%)	20 (33.33%)	9 (45.00%)	
	C	N (%)	26 (43.33%)	6 (30.00%)	
Helkimo Ai	0	N (%)	54 (90.00%)	14 (70.00%)	*p* = 0.049
	I	N (%)	5 (8.33%)	6 (30.00%)	
	II	N (%)	1 (1.67%)	0 (0.00%)	
Helkimo Di	0	N (%)	46 (76.67%)	9 (45.00%)	*p* = 0.01
	I	N (%)	12 (20.00%)	11 (55.00%)	
	II	N (%)	2 (3.33%)	0 (0.00%)	
	III	N (%)	0 (0.00%)	0 (0.00%)	

**Table 5:** Results of statistical analysis of PD, CAL, and mSBI indices for the studied groups in the initial examination.

Quantitative Variables			Group		Mann–Whitney U Test
			Study	Control	
PD		N (%)	45 (100%)	15 (100%)	*p* = 0.017
		Mean	1.82	2.32	
		Std. Deviation	0.63	0.85	
		Median	1.70	2.10	
		Range	1.00–3.10	1.30–3.50	
CAL		N (%)	45 (100%)	15 (100%)	*p* = 0.344
		Mean	2.91	2.78	
		Std. Deviation	1.48	1.59	
		Median	2.70	2.20	
		Range	0.00–7.90	0.80–5.90	
mSBI	>50%	N (%)	20 (44.44%)	5 (33.33%)	*p* = 0.362
		Mean	93.14	73.18	
		Std. Deviation	13.37	19.57	
	50–20%	N (%)	11 (24.44%)	6 (40.00%)	
		Mean	27.07	32.88	
		Std. Deviation	7.33	9.98	
	19–10%	N (%)	3 (6.67%)	3 (20.00%)	
		Mean	14.25	13.54	
		Std. Deviation	4.18	4.00	
	<10%	N (%)	11 (24.44%)	1 (6.67%)	
		Mean	0.64	0.00	
		Std. Deviation	2.11	-	
Mean mSBI		N (%)	45 (100%)	15 (100%)	
		Mean	49.12	40.25	
		Std. Deviation	42.00	28.75	
		Median	32.42	30.75	
		Range	0.00–100.00	0.00–100.00	

**Table 6:** Results of statistical analysis of the MCW parameter and radiomorphometric indices (MCI, PMI) for the studied groups in the initial examination.

Quantitative Variables			Group		Mann–Whitney U Test/Fisher’s Exact Test *
			Study	Control	
MCW		N (%)	60 (100%)	20 (100%)	*p* = 0.471
		Mean	4.60	4.58	
		Std. Deviation	1.31	1.38	
		Median	4.83	4.59	
		Range	1.40–7.69	2.06–6.89	
MCI	C1	N (%)	9 (15.00%)	5 (25.00%)	*p* = 0.33 *
	C2	N (%)	33 (55.00%)	12 (60.00%)	
	C3	N (%)	18 (30.00%)	3 (15.00%)	
PMI		N (%)	60 (100%)	20 (100%)	*p* = 0.367
		Mean	0.3156	0.3361	
		Std. Deviation	0.1035	0.1223	
		Median	0.3281	0.3304	
		Range	0.08–0.62	0.16–0.71	

* Results with an asterisk refer to Fisher’s exact test.

**Table 7:** Results of the Statistical Analysis of Risk Factors for the Investigated Groups in the First Study.

Quantitative/Qualitative Variables			Group		Mann–Whitney U Test/Fisher’s Exact Test *
			Study	Control	
Frequency of Brushing	0	N (%)	7 (11.67%)	1 (5.00%)	*p* = 0.011
	1	N (%)	19 (31.67%)	3 (15.00%)	
	2	N (%)	26 (43.33%)	9 (45.00%)	
	3	N (%)	8 (13.33%)	7 (35.00%)	
Additional Oral Hygiene Products	Yes	N (%)	19 (31.67%)	14 (70.00%)	*p* < 0.001 * OR: 0.20 (95% CI: 0.05–0.67)
	No	N (%)	41 (68.33%)	6 (30.00%)	
Glycocorticosteroids	Yes	N (%)	5 (8.33%)	0 (0.00%)	*p* = 0.32 * OR: 0.00 (95% CI: 0.00–3.28)
	No	N (%)	55 (91.67%)	20 (100.00%)	
Cigarette Smoking	Yes	N (%)	7 (11.67%)	4 (20.00%)	*p* = 0.45 * OR: 1.88 (95% CI: 0.36–8.57)
	No	N (%)	53 (88.33%)	16 (80.00%)	
Alcohol	Yes	N (%)	24 (40.00%)	13 (65.00%)	*p* = 0.07 * OR: 2.75 (95% CI: 0.87–9.42)
	No	N (%)	36 (60.00%)	7 (35.00%)	
Denture	None	N (%)	20 (33.33%)	2 (10.00%)	*p* = 0.06 *
	Fixed	N (%)	11 (18.33%)	8 (40.00%)	
	Removable	N (%)	29 (48.33%)	10 (50.00%)	

* Results with an asterisk refer to Fisher’s exact test.

**Table 8:** The results of the statistical analysis of DMFT, OHI-S, PD, CAL, mSBI, Helkimo Ai, Helkimo Di, MCI, PMI, Eichner’s classification, and MCW parameter for the studied groups in the second study after 6 months.

Quantitative/Qualitative Variables				Group		Mann–Whitney U Test/Fisher’s Exact Test *
				Study	Control	
OHI-S Mean			N (%)	45 (100%)	15 (100%)	*p* < 0.001
			Mean	1.89	0.98	
			Std. Deviation	0.98	0.48	
			Median	2.00	1.00	
			Range	0.00–4.16	0.50–2.00	
Eichner		A1	N (%)	0 (0.00%)	0 (0.00%)	*p* = 0.65 *
		A2	N (%)	1 (1.67%)	0 (0.00%)	
		A3	N (%)	13 (21.67%)	5 (25.00%)	
		B1	N (%)	7 (11.67%)	5 (25.00%)	
		B2	N (%)	5 (8.33%)	1 (5.00%)	
		B3	N (%)	3 (5.00%)	2 (10.00%)	
		B4	N (%)	5 (8.33%)	1 (5.00%)	
		C1	N (%)	3 (5.00%)	1 (5.00%)	
		C2	N (%)	8 (13.33%)	0 (0.00%)	
		C3	N (%)	15 (25.00%)	5 (25.00%)	
Eichner		A	N (%)	14 (23.33%)	5 (25.00%)	*p* = 0.57 *
		B	N (%)	20 (33.33%)	9 (45.00%)	
		C	N (%)	26 (43.33%)	6 (30.00%)	
Helkimo Ai		0	N (%)	52 (86.67%)	13 (65.00%)	*p* = 0.046 *
		I	N (%)	8 (13.33%)	7 (35.00%)	
		II	N (%)	0 (0%)	0 (0.00%)	
Helkimo Di		0	N (%)	43 (71.67%)	9 (45.00%)	*p* = 0.046 *
		I	N (%)	16 (26.67%)	11 (55.00%)	
		II	N (%)	1 (1.67%)	0 (0.00%)	
		III	N (%)	0 (0.00%)	0 (0.00%)	
MCW			N (%)	60 (100%)	20 (100%)	*p* = 0.334
			Mean	4.73	4.56	
			Std. Deviation	1.34	1.38	
			Median	4.91	4.61	
			Range	1.59–8.34	2.03–6.92	
MCI	C1		N (%)	15 (25.00%)	5 (25.00%)	*p* = 0.62 *
	C2		N (%)	29 (48.33%)	12 (60.00%)	
	C3		N (%)	16 (26.67%)	3 (15.00%)	
PMI			N (%)	60 (100%)	20 (100%)	*p* = 0.489
			Mean	0.3245	0.3338	
			Std. Deviation	0.1050	0.1218	
			Median	0.3328	0.3262	
			Range	0.09–0.64	0.16–0.70	
PD			N (%)	45 (100%)	15 (100%)	*p* = 0.020
			Mean	1.87	2.35	
			Std. Deviation	0.65	0.83	
			Median	1.70	2.20	
			Range	1.00–3.30	1.30–3.50	
CAL			N (%)	45 (100%)	15 (100%)	*p* = 0.376
			Mean	2.98	2.84	
			Std. Deviation	1.49	1.59	
			Median	2.80	2.20	
			Range	0.00–8.00	0.90–5.90	
mSBI	>50%		N (%)	25 (55.56%)	2 (13.33%)	*p* = 0.003
			Mean	91.46	60.17	
			Std. Deviation	16.51	9.66	
	50–20%		N (%)	10 (22.22%)	7 (46.67%)	
			Mean	33.70	31.64	
			Std. Deviation	8.02	10.36	
	19–10%		N (%)	4 (8.89%)	2 (13.33%)	
			Mean	14.06	15.12	
			Std. Deviation	3.21	5.48	
	<10%		N (%)	6 (13.33%)	4 (26.67%)	
			Mean	1.04	4.42	
			Std. Deviation	2.55	3.06	
mSBI Mean			N (%)	45 (100%)	15 (100%)	
			Mean	59.69	25.98	
			Std. Deviation	39.35	19.74	
			Median	60.50	22.00	
			Range	0.00–100.00	0.00–67.00	

* Results with an asterisk refer to Fisher’s exact test.

**Table 9:** Results of statistical analysis of the changes in the values of indicators in two studies comparatively in two groups: test and control.

Independent Variable	Dependent Variable	Logistic Regression (*p*)	Coef, 95% CI	ROC AUC	Mann–Whitney U Test
Change in OHI-S	Group	0.003	1.2139 (0.415; 2.012)	0.75	*p* < 0.001
Change in Helkimo Ai	Group	0.500	0.0000 (−1.132; 1.13)	0.50	*p* = 0.420
Change in Helkimo Di	Group	0.484	0.5108 (−0.921; 1.942)	0.52	*p* = 0.339
Change in CAL	Group	0.057	5.0054 (−0.143; 10.154)	0.46	*p* = 0.286
Change in PD	Group	0.065	5.4375 (−0.340; 11.215)	0.48	*p* = 0.400
Change in mSBI	Group	0.002	0.0905 (0.032; 0.149)	0.84	*p* = 0.001
Change in MCW	Group	0.001	6.7605 (2.756; 10.765)	0.82	*p* < 0.001

## Data Availability

The data is contained within the article, further inquiries can be directed to the corresponding authors.
